# Fly ash upcycling to functional silica nanomaterials: insights into synthetic strategies towards efficient adsorbents for water purification

**DOI:** 10.1039/d5ra08626d

**Published:** 2026-01-02

**Authors:** Miguel S. Ruiz, Cristian Tunsu, Fredric G. Svensson, Ani Vardanyan

**Affiliations:** a Department of Molecular Sciences, Swedish University of Agricultural Sciences P.O. Box 7015 75007 Uppsala Sweden ani.vardanyan@slu.se; b EasyMining Services AB Ultunaallén 2A 756 51 Uppsala Sweden; c Department of Materials Science and Engineering, The Ångström Laboratory, Uppsala University Uppsala Box 35 751 03 Sweden

## Abstract

The transformation of fly ash into high-value nanomaterials presents a sustainable route for waste valorization. In this study, silica nanoparticles were synthesized from fly ash using three different methods: acid-precipitation of dense silica, surfactant-templated sol–gel synthesis for mesoporous silica, and a one-pot thermal activation process integrating silica extraction and nanoparticle formation. The resulting nanomaterials were functionalized with amine ligand to enhance their affinity toward anionic pharmaceutical pollutants in water. Physicochemical characterization confirmed successful silica formation and surface modification. The mesoporous silica exhibited a specific surface area of 620 m^2^ g^−1^ and well-defined pore architecture, in contrast to the denser or less-ordered structures obtained by the other two approaches. The materials were evaluated for adsorption of diclofenac, a commonly detected anionic water pollutant. Uptake experiments revealed that both the kinetics and capacity of adsorption were influenced by the degree of functionalization and pore accessibility. The surfactant-templated mesoporous silica displayed the most effective removal, achieving rapid initial adsorption and high capacity. This study offers a side-by-side comparison of scalable pathways for producing high-performance adsorbents from industrial waste. The findings provide insight into how synthetic strategy selection can tailor physicochemical properties, guiding the design of fly ash-derived materials for environmental remediation and other functional applications.

## Abstract 2

The transformation of fly ash into high-value nanomaterials presents a sustainable route for waste valorization.

## Graphical abstract

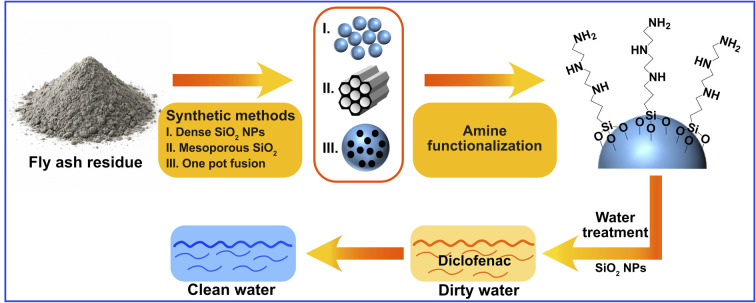

## 1 Introduction

Fly ash (FA), a byproduct of industrial waste incineration, is rich in silicon, aluminum, and calcium, making it an attractive low-cost resource for producing value-added materials. Composed primarily of amorphous glassy phases along with crystalline quartz and mullite, FA has been widely explored as a silica source for advanced materials, supporting circular economy efforts and reducing environmental impact.^1,2^

Sol–gel chemistry offers a versatile route for converting FA-derived silica into nanostructured materials with tunable properties.^3,4^ Since the pioneering work of Stöber *et al.* (1968) on monodisperse silica particles, various synthesis methods have emerged, enabling precise control over particle size, morphology, and porosity.^5^ Among these, dense and mesoporous silica nanoparticles are especially attractive for applications such as catalysis, adsorption, and controlled release, due to their high surface area and modifiable surfaces.^6–10^

Synthesizing silica nanomaterials from FA typically involves two key steps: silicon extraction and material synthesis. In the extraction step, alkali dissolution or alkali fusion methods are commonly employed to convert the silicon content into soluble silicate species.^11,12^ During alkali dissolution, amorphous silica phases in FA react with sodium hydroxide to form soluble silicate ions (SiO_3_^2−^), while the more stable crystalline components (*e.g.*, quartz, mullite) remain largely unreacted.^13^ This method is advantageous due to its low energy requirements and minimal equipment corrosion but is inherently limited by the fraction of amorphous silica in the FA. Reported extraction efficiencies vary, with up to 46.6% achieved using NaOH alone and improvements up to 54.4% when introducing ultrasonic waves in the extraction process.^14,15^

In contrast, alkali fusion involves mixing FA with solid sodium hydroxide or other auxiliaries (*e.g.*, Na_2_CO_3_, CaCO_3_, NH_4_F) and subjecting the mixture to high temperatures to disrupt stable crystalline phases.^16–18^ This thermal activation transforms Si- and Al-containing phases into soluble sodium silicate and sodium aluminate, which then are dissolved by water, followed by filtration.^19^

The extracted sodium silicate solution can then be used to synthesize either dense silica nanoparticles (typically *via* acid precipitation) or mesoporous silica using surfactant-templated sol–gel methods with agents like Pluronic 123 or CTAB. Several studies have demonstrated the successful preparation of such materials from FA-derived precursors. Yadav and Fulekar (2018) synthesized nanosilica from FA *via* chemical and biological routes, yielding 20–70 nm spherical aggregates with 90–96% purity, while in another study by Liang *et al.* (2020) spherical nanosilica particles with average size of 20–40 nm were produced through sol–gel processing of FA with ∼93% purity.^11,20^

In addition to the conventional two-step methods, one-pot synthesis approaches have also been explored. These strategies integrate silica extraction and nanoparticle formation into a single step, offering a simplified workflow and potentially greater process efficiency but may pose challenges in controlling particle morphology and surface functionalization. Imoisili and Jen (2022), for instance, employed a microwave-assisted sol–gel method to produce template-free nanosilica directly from South African FA, demonstrating the feasibility of one-pot processes under mild conditions.^21^

In this study, we compare three synthetic strategies for producing silica nanoparticles from FA: acid-precipitated dense silica, surfactant-templated mesoporous silica, and a one-pot synthesis method. To enhance their functionality, the synthesized materials were further modified with amine groups and tested for their potential in removing the organic pollutant diclofenac (DFC) from water. This study not only highlights the potential of FA-derived materials in water treatment applications but also serves as a valuable reference for selecting efficient and sustainable synthetic strategies for future applications.

## 2 Materials and methods

### 2.1 Materials

FA from mono-incineration of sewage sludge in a fluidized bed reactor (European provenience) was used. For the synthetic procedures, the following reagents have been used: sodium hydroxide (pellets), CAS:1310-73-2, ScharLab (Spain), sodium carbonate, CAS:497-19-8, Merck (Germany), cetyltrimethylammonium bromide (CTAB), CAS:57-09-0, Sigma-Aldrich (Germany), *N*^1^-(3-trimethoxysilylpropyl) diethylenetriamine (TMSPDETA), CAS:35141-30-1, Sigma-Aldrich (Germany), diclofenac sodium salt (DFC), CAS:15307-79-6, Sigma-Aldrich (Germany), poly(ethylene glycol)-*block*-poly(propylene glycol)-*block*-poly(ethylene glycol) (Pluronic 123), CAS:9003-11-6, Sigma-Aldrich (Germany), hydrochloric acid 37%, CAS:7647-01-0, Sigma-Aldrich (Germany), nitric acid, CAS:7697-37-2, Sigma-Aldrich (Germany), ethanol, CAS:64-17-5, Solveco (Sweden), toluene, CAS:108-88-3, Sigma-Aldrich (Germany).

### 2.2 Methods

#### 2.2.1 Conversion of fly ash into silicate precursors

Chemical treatment of FA began with an acid leaching using hydrochloric acid to selectively remove calcium phosphate, hematite and other impurities, yielding a silicate-rich residue with a Si content of 34.3 wt%. Detailed elemental compositions of the original ash and FA residue are provided in the SI (Tables S1 and S2).

For silica extraction, 5 g of FA residue was mixed with 8 M NaOH solution, maintaining a solid-to-liquid ratio of 1 : 5, and heated to 90 °C for 1.5 hours under continuous stirring at 500 rpm in a 100 mL round-bottom flask equipped with a condenser. After the reaction, the mixture was cooled to room temperature, and the residue was separated by centrifugation at 7000×*g* for 10 minutes, followed by filtration using Whatman filter paper. The resulting sodium silicate solution (SSS) was stored at 4 °C until further use.

#### 2.2.2 Synthesis of silica nanomaterials

##### 2.2.2.1 Sol–gel approach to dense silica nanoparticles: D_SiO_2_

The synthesis of D_SiO_2_ nanoparticles was adapted from previously reported procedures with some modifications.^11^ Typically, 50 mL of SSS was mixed with 10 mL of ethanol in a 100 mL beaker and heated in a water bath at 50 °C. Concentrated nitric acid was added dropwise to adjust the pH to either 2 or 7, both of which resulted in the immediate formation of silica nanoparticles. In both cases, the reaction mixture was maintained for an additional hour to allow particle maturation. The nanoparticles were then collected by centrifugation at 7000×*g* for 10 minutes and washed three times each with ethanol and Milli Q water to remove residual sodium silicate. Finally, the nanoparticles were dried overnight under nitrogen atmosphere.

##### 2.2.2.2 Synthesis of mesoporous silica nanoparticles: M_SiO_2_

Mesoporous silica nanoparticles (M_SiO_2_) were prepared following the method described by Kobylinska *et al.* (2022) with modifications.^22^ The SSS used for this synthesis was obtained as described in Section 2.2.1. For the synthesis, 4 g of the surfactant Pluronic 123 was dissolved in 60 mL of deionized water, and 38 mL of concentrated HCl was added to the solution under continuous stirring. The mixture was heated in a water bath at 40 °C and stirred for 30 minutes to ensure complete dissolution of the surfactant. Subsequently, 40 mL of the SSS was added dropwise to the mixture and stirred under the same conditions for 2 hours. Following this, the reaction mixture was heated to 80 °C and maintained for 20 hours to allow particle formation and structural development.

The synthesized nanoparticles were collected by centrifugation at 7000×*g* for 10 minutes and subjected to template removal. This was achieved by boiling the particles in acidified ethanol for 3 hours, repeated four times with fresh ethanol for each cycle. The nanoparticles were then vacuum dried sequentially: 30 minutes at room temperature, 30 minutes at 50 °C, and 3 hours at 100 °C, yielding mesoporous silica nanoparticles.

##### 2.2.2.3 1-Pot synthesis of mesoporous silica nanoparticles: 1 pot_M_SiO_2_

Mesoporous silica nanoparticles were synthesized directly from FA residue using a one-pot fusion method, eliminating the need for prior silica extraction.^19^

In the first step, crystalline transformation of FA residue was achieved by mixing and grinding 2 g of FA with 2.4 g of sodium carbonate (Na_2_CO_3_), maintaining a mass ratio of 1.2 (Na_2_CO_3_ to FA residue). The homogeneous mixture was then transferred to a muffle furnace and calcined at 850 °C for 1.5 hours to convert stable mineral phases into more reactive forms.

Separately, 20 mL of concentrated HCl was diluted to 80 mL with deionized water, and 0.6 g of CTAB was dissolved in the solution under continuous stirring until fully dissolved. Following calcination, 2 g of the calcined mixture was added to the CTAB-HCl solution and stirred at 400 rpm at room temperature for 1 hour to facilitate mesoporous structure formation.

After the reaction, the supernatant was removed by centrifugation at 7000×*g* for 10 minutes. The resulting solid was then calcined at 550 °C for 4 hours to remove the CTAB template, yielding mesoporous silica nanoparticles.

#### 2.2.3 Characterization

The morphology of FA residue and the synthesized silica nanomaterials was examined by scanning electron microscopy (SEM) using Hitachi (Tokyo, Japan) Flex-SEM 1000 environmental SEM. The instrument was operated at an acceleration voltage of 5 kV with a spot size of 20 and a working distance of 5 mm. Elemental analysis of surfaces were performed using SEM with energy dispersion spectroscopy (EDS) with the combination of Hitachi (Tokyo, Japan) Flex-SEM 1000 environmental SEM and AZtecOneXplore EDS detector by Oxford instruments (UK). For each sample at least five random points were analyzed under 20 kV accelerating voltage, spot size 50, and 10 mm working distance. The relative elemental proportions were determined from the average results of these measurements.

Powder X-ray Diffraction (PXRD) patterns were collected using a Bruker D8 QUEST ECO diffractometer equipped with a Proton III Area Detector and graphite-monochromated Mo-Kα (*λ* = 0.71073 Å) radiation source. The diffraction data were analyzed using the EVA-12 software package.

The concentration of DFC and its degradation kinetic were monitored by UV-vis spectroscopy using a Multiskan Sky High (Thermo Fisher Scientific, Waltham, MA, USA) spectrophotometer with quartz cuvettes. Spectra were recorded in the 200–600 nm range to determine the wavelength of maximum absorbance. Prior to analysis, all samples were passed through 0.2 µm cellulose filters to remove suspended composite materials.

Nitrogen adsorption–desorption isotherms were measured at −196 °C using a Micromeritics ASAP 2020 surface area and porosity analyzer (Norcross, GA, USA) to determine specific surface area and pore volume. Prior to analysis, the samples were dried in an oven at 120 °C for 2 h, stored in sealed containers within a desiccator, and subsequently degassed under vacuum at 120 °C for 2 h.

Fourier transform infrared (FTIR) spectra of FA, synthesized nanomaterials and the functionalized nanoparticles were collected on a PerkinElmer spectrum 100 spectrometer using KBr pellets. Thermogravimetric analysis (TGA) was performed on a PerkinElmer Pyris 1 (Waltham, MA, USA) in air, heating from 25 to 900 °C at 5 °C min^−1^ rate.

#### 2.2.4 Functionalization of silica nanoparticles with TMSPDETA ligand

All three synthesized silica nanomaterials: D_SiO_2_, M_SiO_2_ and 1 pot_M_SiO_2_, were functionalized with a 3-amino organic ligand to enhance their surface properties for water treatment applications. The functionalization protocol was adapted from procedures previously developed in our research group.^23–25^

In a typical procedure, 500 mg of silica nanoparticles were dispersed in 20 mL of toluene, followed by the addition of 1 mL of the organosilane-containing TMSPDETA ligand. The mixture was refluxed for 24 hours under an inert nitrogen atmosphere to promote covalent bonding between the silica surface and the ligand.

After the reaction, the functionalized nanoparticles were collected by centrifugation at 7000×*g* for 10 minutes. The particles were subsequently washed twice with toluene and twice with ethanol to remove unreacted ligand and byproducts. Finally, the nanoparticles were dried overnight under nitrogen atmosphere.

#### 2.2.5 Adsorption of organic pollutant from water

Adsorption experiments were performed using 50 mg of each adsorbent material: D_SiO_2__3NH, M_SiO_2__3NH, and 1 pot_M_SiO_2__3NH. DFC was used as a model pollutant. A stock solution of DFC (100 µg mL^−1^) was prepared in Milli Q water and diluted to generate calibration standards at concentrations of 1, 2, 4, 6, 8, and 10 µg mL^−1^ (Fig. S6). For adsorption isotherms, additional DFC solutions were prepared at 1, 2, 5, 10 and 20 µg mL^−1^. Each adsorption experiment was conducted in a 15 mL Falcon tube containing 5 mL of the DFC solution and 50 mg of adsorbent. The tubes were placed on an orbital shaker and agitated at room temperature for 24 h to reach equilibrium.

For kinetic studies, the same experimental conditions were used with an initial DFC concentration of 5 µg mL^−1^. Samples were collected at predefined time intervals. At each time point, tubes were centrifuged at 7000×*g* for 10 min, and 1 mL of supernatant was withdrawn and transferred to a quartz cuvette for UV-vis analysis. Absorbance spectra were recorded between 240 and 340 nm, with the characteristic DFC absorption peak observed at ∼273 nm. After each measurement, the 1 mL aliquot was returned to the respective tube to maintain constant volume throughout the experiment.

## 3 Results and discussion

### 3.1 Conversion of fly ash into silicate precursors

The conversion of FA residue into soluble silicate precursors was successfully achieved through alkali dissolution. The composition of the FA residue before and after alkali dissolution was determined by SEM-EDS analysis and is presented in Table S3. The EDS results indicate that the predominant elements are Si, Al, O, and C, with percentage contributions of 17.9%, 2.4%, 57.1%, and 15.03%, respectively, consistent with a matrix dominated by the corresponding oxides SiO_2_ and Al_2_O_3_. After the desilication step, the silicon content decreased by approximately 58%.

Following this dissolution, the elemental composition of the resulting sodium silicate solution (SSS) is shown in Table S4. As expected, sodium originates primarily from the NaOH, whereas silicon is sourced from the FA residue. Minor impurities, including phosphorus and nitrogen, were also transferred from the residue into the SSS. The detection of dissolved silicon confirms the formation of sodium silicate.

The EDS spectra of the as-synthesized D_SiO_2_ nanoparticles (Fig. S1) show peaks for Si, O, C, Na and Fe. The presence of Si and O signals confirm the formation of silica from the FA residue. The presence of residual Na and trace Fe is due to the impurities associated with incomplete washing, while C signal arises from the FA residue and adhesive carbon tape.

### 3.2 Characterization of SiO_2_ nanomaterials

#### 3.2.1 Particle morphology

**Fig. 1:**
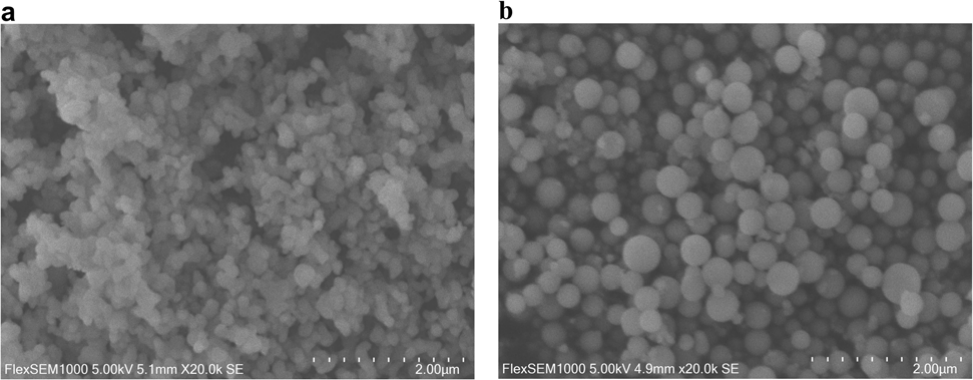
SEM images of synthesized D_SiO_2_ NPs from FA residue at pH = 2 (a) and pH = 7 (b).

The size and morphology of the synthesized SiO_2_ nanomaterials were characterized using scanning electron microscopy (SEM). For the D_SiO_2_ nanoparticles, the SEM images showed that those synthesized at pH 2 exhibited a more monodisperse, spherical morphology with an average diameter of approximately 200 nm (Fig. 1a). In contrast, nanoparticles synthesized at pH 7 displayed a broader size distribution, with larger spherical particles ranging from 400 to 500 nm in diameter (Fig. 1b). The formation of these different particle morphologies was highly dependent on the reaction conditions investigated in this work. At low pH (1–2), rapid hydrolysis produced a high supersaturation of silicate species, leading to burst nucleation and the formation of smaller, more uniform particles, as monomers were rapidly depleted. In contrast, at neutral pH (7), the hydrolysis step proceeds more slowly, generating monomeric silica at a reduced rate. Under these conditions, nucleation occurs more gradually, while existing nuclei have more time to grow by condensation, resulting in larger spherical particles and a broader size distribution. This behavior is consistent with classical sol–gel theory, which describes the pH-dependent balance between hydrolysis and condensation processes,^26^ and with prior studies demonstrating strong pH control over silica polymerization and particle growth kinetics.^27^

In the case of mesoporous silica nanoparticles, synthesized using both the one-pot method and the two-step method (involving sodium silicate extraction), the particles exhibited less organized structures, and impurities were observed in both samples (Fig. S2). For M_SiO_2_ silica sample, the particles appeared as non-uniform agglomerates with sizes ranging between 300 and 500 nm. In contrast, the 1 pot_M_SiO_2_ synthesis produced smaller, more organized, and round-shaped particles, with sizes ranging from 150 to 200 nm. Additionally, both samples contained large, plate-like flakes, which were likely impurities originating from the sodium silicate solution and, in the case of the one-pot synthesis, potentially from unreacted FA residue. This was confirmed by EDS analysis which identified trace amounts of Al, N, Na, Fe and P in both the samples (Fig. S3 and S4).

#### 3.2.2 Pore structure analysis

**Fig. 2:**
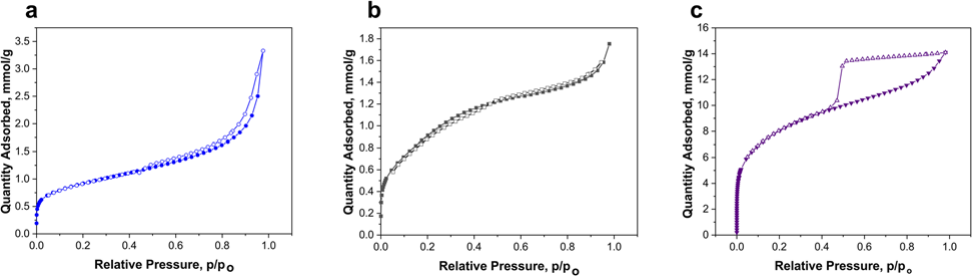
Nitrogen adsorption–desorption isotherm plots of silica nanomaterials synthesized through acid-precipitation: D_SiO_2_ (a), one-pot thermal activation process: 1 pot_M_SiO_2_ (b) and, surfactant-templated sol–gel synthesis: M_SiO_2_ (c).

The nitrogen adsorption–desorption isotherms for the three synthesized silica samples are presented in Fig. 2, and the corresponding BET surface area, BJH pore area, and average pore diameter are summarized in Table 1.

**Table 1:** Physicochemical properties of synthesized SiO_2_ nanomaterials

Sample	Surface area (m^2^ g^−1^)	BJH pore area^a^ (m^2^ g^−1^)	BJH average pore width^a^ (Å)
M_SiO_2_	623	467	33.8
D_SiO_2_	68	61	36.1
1 pot_M_SiO_2_	75	52	81.1

a Pore area and average pore width were determined from the desorption part of the isotherm.

The analysis reveals clear distinctions in textural properties that directly reflect the influence of the chosen synthetic strategies.

The M_SiO_2_ nanoparticles, synthesized *via* a two-step surfactant-templated sol–gel approach using Pluronic P123, displayed a type IV isotherm with an H4-type hysteresis loop, characteristic of mesoporous materials with narrow slit-like pores, in accordance with IUPAC and de Boer classifications.^28^ This sample exhibited a high BET surface area of 623 m^2^ g^−1^ and a BJH pore area of 467 m^2^ g^−1^, indicating that most of the surface area is derived from accessible mesopores. The average BJH pore width was 34 Å, confirming a uniform mesoporous structure. These values align well with the expected performance of mesoporous silica synthesized using triblock copolymer templates and suggest successful template removal without significant pore collapse. The well-developed mesoporosity of this material contributes to its superior adsorption performance, as discussed later.

In contrast, the D_SiO_2_ nanoparticles, synthesized by acid precipitation from extracted sodium silicate solution, exhibited a type II isotherm with no pronounced hysteresis loop. This isotherm profile is typical for non-porous or macroporous materials, where surface adsorption occurs on the external surfaces of dense particles rather than within internal pores. The BET surface area was 68 m^2^ g^−1^, and the BJH pore area was 61 m^2^ g^−1^, which are consistent with dense silica particles reported in the literature.^23,29^ Although the calculated BJH pore width was 36 Å, this likely reflects interparticle voids or minor surface roughness rather than a true mesoporous network.

The relatively low surface area and absence of mesoporosity support the classification of this material as structurally dense.

The 1 pot_M_SiO_2_ sample, synthesized *via* a one-pot thermal activation and condensation method using CTAB as a structure-directing agent, also showed a type II isotherm, though with a larger BJH pore width of 81 Å, suggesting the formation of broader pores or interparticle gaps.

However, the BET surface area (75 m^2^ g^−1^) and BJH pore area (52 m^2^ g^−1^) were considerably lower than those of the M_SiO_2_ sample. This indicates that despite the intent to form mesoporous structures, the actual pore accessibility and development were limited. The lower porosity may be attributed to the less controlled conditions inherent to the one-pot method, including potential incomplete dissolution of the FA residue or interference from residual mineral phases and impurities. Additionally, partial pore collapse during drying or calcination may have contributed to the reduced surface area.^30^

These results clearly demonstrate that the surfactant-assisted sol–gel synthesis yields the most well-defined and accessible mesoporous structure, while acid precipitation produces dense particles, and the one-pot synthesis results in intermediate properties with limited porosity development.

#### 3.2.3 Analysis of FTIR and XRD

**Fig. 3:**
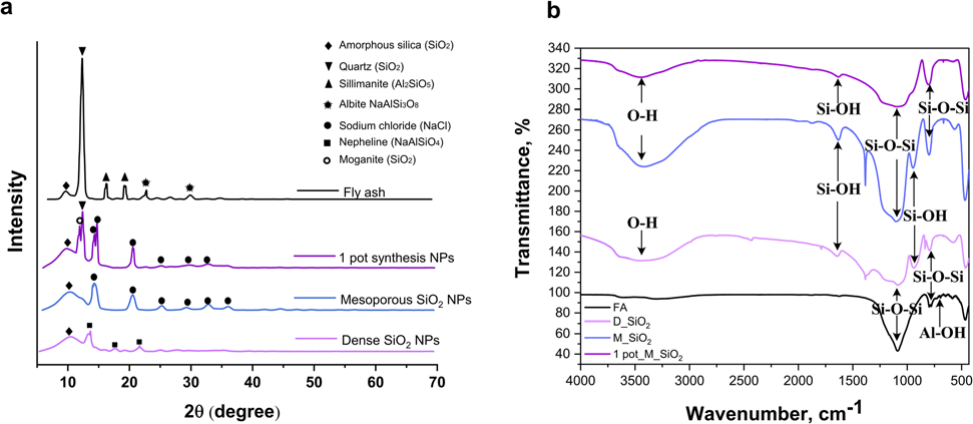
XRD patterns (a) and FTIR spectra (b) of FA residue before and after SiO_2_ nanomaterials' synthesis.

The composition of FA residue and synthesized silica nanomaterials were characterized based on X-ray diffraction (XRD) patterns presented in Fig. 3. The major crystalline phases identified in FA residue were quartz (SiO_2_, 2*θ* = 12.35°), sillimanite (Al_2_SiO_5_, 2*θ* = 16.25° and 19.25°) and albite (NaAlSi_3_O_8_, 2*θ* = 22.75° and 29.85°). The diffraction peak at 2*θ* = 9.55° indicated the presence of an amorphous silica fraction.^14^ After the synthesis of silica nanoparticles, most of the crystalline peaks disappeared, leaving primarily a broad peak around 2*θ* ≈ 9–10°, characteristic of amorphous silica. This transformation was observed for both the dense and mesoporous silica nanoparticles synthesized *via* the two-step process, where alkali extraction dissolved amorphous silica and partially attacked albite, followed by acid precipitation or surfactant-assisted sol–gel condensation. In both cases, residual sodium chloride peaks were detected, likely originating from incomplete removal of salts during the washing steps. In the 1 pot_M_SiO_2_ nanoparticles, a predominantly amorphous structure was also obtained, however, weak diffraction peaks corresponding to quartz and moganite remained, suggesting the presence of residual crystalline impurities. These likely originated from incomplete dissolution or transformation during the direct synthesis process. According to previous literature during calcination with sodium carbonate, stable phases like quartz and mullite react to form sodium silicate (Na_2_SiO_3_), sodium aluminum oxide silicate (NaAlSiO_4_) and nepheline (Na_2.8_K_0.6_Ca_0.2_Al_3.8_Si_4.2_O_16_) with the product distribution influenced by the FA composition.^19,31^ In our case, the conversion of albite and quartz to soluble sodium silicate provided the main silica source for nanoparticle formation, while small amounts of unconverted quartz were retained.

Overall, the XRD results confirm the effective transformation of crystalline phases in FA residue into predominantly amorphous silica, with only minor residual crystallinity detected in the 1 pot_M_SiO_2_.

### 3.3 Functionalization of silica nanoparticles

**Fig. 4:**
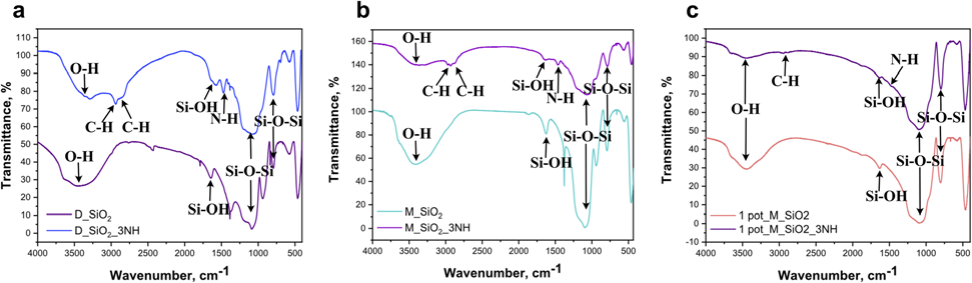
FTIR spectra of synthesized nanoparticles before and after grafting with TMSPDETA ligand: (a) D_SiO_2_, (b) M_SiO_2_ and (c) 1 pot_SiO_2_ NPs.

To assess surface functionalization, FTIR analysis were performed before and after ligand grafting. The FTIR spectra revealed characteristic bands consistent with silica structures as well as additional peaks corresponding to functionalized silica nanoparticles, confirming successful ligand attachment in the synthesized samples (Fig. 3b and 4a–c). For comparison, the FTIR spectrum of the FA residue exhibited a distinct peak at 730 cm^−1^, attributed to Al–OH groups.^32^ This band was absent in all synthesized silica nanoparticles, consistent with the removal of alumina-containing phases during processing and confirming the formation of purified SiO_2_ prior to functionalization.

The IR spectrum of three silica nanomaterials showed prominent peaks at 470 cm^−1^, 800 cm^−1^, and 1080 cm^−1^, corresponding to *δ*(Si–O–Si), *ν*(Si–O–Si), and *ν*_as_(Si–O–Si) vibrations, respectively.^23,32^ A broad band around 3500 cm^−1^and a peak at 1640 cm^−1^ indicated the presence of residual hydroxyl groups *ν*_as_(Si–OH) and *δ*(O–H) vibrations.^33–35^

After functionalization with amine ligands (D_SiO_2__3NH, M_SiO_2__3NH, 1pot_M_SiO_2__3NH), additional peaks were observed at 2930 cm^−1^ and 2980 cm^−1^, corresponding to *ν*(C–H) stretching vibrations from the alkyl chains of the ligands, as well as a peak at 1465 cm^−1^ attributed to the amine group.^23,24,36^ These new bands confirmed the successful grafting of ligands onto the silica nanoparticles. However, the 1 pot_M_SiO_2__3NH exhibited smaller peaks at 2930 cm^−1^, 2980 cm^−1^, and 1465 cm^−1^, suggesting a lower extent of ligand grafting which may be attributed to residual surface impurities and less controlled reaction conditions, which can reduce the availability of accessible silanol groups for effective ligand attachment.

Surface functionalization was further confirmed through TGA analysis to assess the thermal stability and degree of ligand grafting on the synthesized silica nanoparticles (Fig. S5).

All samples displayed a multistep weight loss profile corresponding to moisture evaporation, residual ethanol and organic ligand decomposition, and combustion.

Weight losses of approximately 1.9–16.1% were observed for all silica nanoparticles between 20–200 °C, corresponding to surface dehydration and evaporation of residual solvents (toluene).^32^ In the 200–600 °C range, further weight losses of around 3.2% for 1 pot_M_SiO_2__3NH NPs, 29.6% for M_SiO_2__3NH NPs, and 25% for D_SiO_2__3NH NPs were recorded, attributed to the thermal decomposition of the grafted amine ligand. Above 600 °C, additional small losses of ∼2.4%, ∼3.8%, and ∼0.8%, respectively, were related to the decarbonization of residual organic fragments. These results confirm successful functionalization of the silica surfaces, with grafted ligand amounts following the trend: M_SiO_2_ >D_SiO_2_ >1 pot_M_SiO_2_ NPs. This is consistent with the FTIR results and can be attributed to a combination of residual inorganic surface impurities and lower density of reactive silanol groups, both of which may limit effective functionalization in the one-pot material (Table S5).

### 3.4 Adsorption experiments

**Fig. 5:**
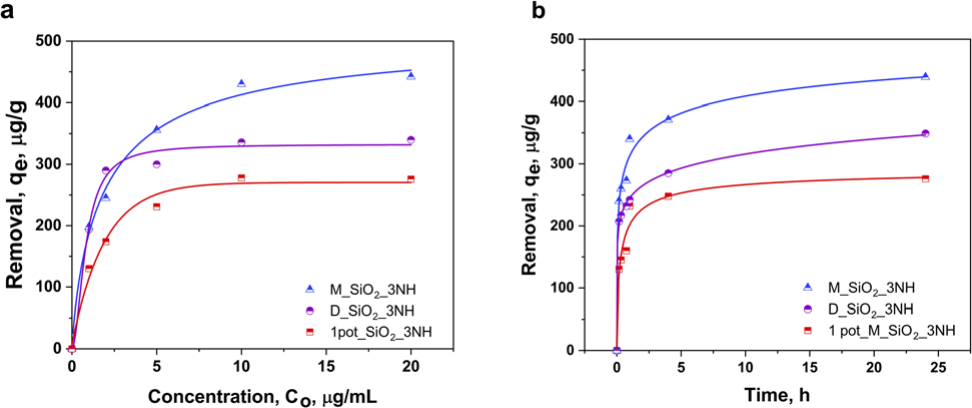
Adsorption of DFC on TMSPDETA-grafted SiO_2_ nanomaterials: (a) Langmuir adsorption isotherms (b) adsorption kinetics of DFC by D_SiO_2_, M_SiO_2_ and 1 pot_M_SiO_2_ NPs.

The synthesized nanomaterials were evaluated for their adsorption performance in the removal of organic pollutants from water. DFC, a commonly detected pharmaceutical contaminant in surface and drinking water, was selected as a model compound. The adsorption capacities of the synthesized materials toward DFC were evaluated at pH 6, yielding maximum uptake values of 278 µg g^−1^ for 1 pot_M_SiO_2_, 340 µg g^−1^ for D_SiO_2_, and 442 µg g^−1^ for M_SiO_2_ (Fig. 5a). These trends correlate well with the degree of surface grafting, which was highest in M_SiO_2_, followed by D_SiO_2_ and 1 pot_M_SiO_2_. The enhanced performance of M_SiO_2_ can be attributed to both a higher density of surface amine functionalities and better preservation of porosity following post-grafting. The equilibrium adsorption data were fitted using the nonlinear Langmuir isotherm model (with the use of OriginPro), which showed excellent agreement with the experimental results, with *R*^2^ values ranging from 0.98 to 0.99 for all materials. The good fit to the Langmuir model indicates monolayer adsorption on energetically uniform surface sites, consistent with the functionalized silica surfaces.

These trends correlate well with the degree of surface grafting, which was highest in M_SiO_2_, followed by D_SiO_2_ and 1 pot_M_SiO2. The enhanced performance of M_SiO_2_ can be attributed to both a higher density of surface amine functionalities and better preservation of porosity following post-grafting. To further assess the role of surface chemistry, adsorption experiments were also performed using the non-functionalized silica nanoparticles at the highest DFC concentration tested (20 µg mL^−1^). Both the dense silica (D_SiO_2_) and the one-pot silica (1 pot_M_SiO_2_) showed negligible adsorption under these conditions, indicating that their surfaces provided no significant affinity toward the anionic DFC. In contrast, the non-functionalized mesoporous silica (M_SiO_2_) exhibited a measurable, yet low, adsorption capacity of approximately 50 µg g^−1^, which is likely attributed to physical adsorption within accessible mesopores rather than to specific chemical interactions. This value is comparable to previously reported adsorption capacities of non-functionalized mesoporous silicas, such as HMS (32–36 µg g^−1^), MCM-41 (33 µg g^−1^), and SBA-15 (34–100 µg g^−1^), indicating that the performance of FA-derived silica aligns well with established materials when no functional groups are present (Table 2).^36–38^

**Table 2:** Reported adsorption capacities of DFC on silica-based materials^a^

Material	Removal, µg g^−1^	Ref.
HMS	32	36
M-HMS	36	36
A-HMS	6	36
SBA-15	34	36
MCM-41	33	36
PAC	41	36
SBA-15	40	37
SBA-15	100	38
D_SiO_2__3NH	340	Our study
M_SiO_2__3NH	442	Our study
1pot_M_SiO_2__3NH	278	Our study

a M-HMS-mercapto-functionalized HMS, A-HMS-amino functionalized HMS, PAC-powdered activated carbon.

Upon functionalization, however, all three silica nanomaterials demonstrated considerably higher adsorption capacities which highlights the effectiveness of TMSPDETA grafting in promoting electrostatic interactions with anionic DFC (Table 2). All experiments were performed at pH 6, under which DFC is negatively charged. Literature reports a point of zero charge of 9.03 for the TMSPDETA ligand, indicating that the adsorbent surfaces were positively charged at the experimental pH.^39^ Therefore, electrostatic attraction between the protonated amine groups on the adsorbents and the anionic DFC likely played a dominant role in the adsorption mechanism.

Kinteic experiments were performed to examine the effect of reaction time on the adsorption of DFC to the functionalized SiO_2_ nanomaterials (Fig. 5b and S7). The results showed that 60–70% of DFC uptake occurred within the first 2 hours, with adsorption gradually reaching equilibrium by 24 hours. This rapid initial uptake suggests strong surface interactions, followed by slower diffusion-limited processes, possibly involving adsorption into pores or onto less accessible internal surfaces.

## 4 Conclusions

This study demonstrates the successful upcycling of FA into functionalized silica nanomaterials and highlights how synthesis route governs the structural properties and adsorption performance of the final materials.

Acid precipitation produced dense silica nanoparticles, surfactant-templated sol–gel processing generated well-defined mesoporous silica, and a one-pot thermal activation method yielded partially porous structures with residual impurities. All materials were successfully grafted with TMSPDETA ligands, with the extent of functionalization following the trend M_SiO_2_ > D_SiO_2_ > 1 pot_M_SiO_2_, consistent with FTIR and TGA analyses.

Adsorption experiments showed that ligand density and pore accessibility were the dominant factors controlling DFC uptake. The mesoporous M_SiO_2__3NH exhibited the highest adsorption capacity (442 µg g^−1^) and fastest kinetics, while D_SiO_2__3NH and 1 pot_M_SiO_2__3NH showed progressively lower performance in line with their lower porosity and functionalization levels. The excellent fit of the adsorption data to the Langmuir model (*R*^2^ = 0.98–0.99) confirms monolayer adsorption on chemically uniform amine-functionalized surfaces.

Overall, this study highlights the importance of pairing suitable extraction and synthesis strategies with targeted surface modification to achieve high-performance FA-derived adsorbents. These insights provide a foundation for designing scalable, low-cost materials for environmental remediation and broader circular-economy applications.

## Author contributions

Miguel S. Ruiz: formal analysis, investigation, writing – review & editing, Cristian Tunsu: resouces, writing – review & editing, investigation, Fredric. G. Svensson: investigation, writing – review & editing, Ani Vardanyan: conceptualization, funding acquisition, supervision, writing – original draft.

## Conflicts of interest

The authors have no competing interests to declare that are relevant to the content of this article.

## Supplementary Material

RA-016-D5RA08626D-s001

## Data Availability

Data supporting this study are included within the article and supplementary information (SI). Supplementary information is available. See DOI: https://doi.org/10.1039/d5ra08626d.
