# Supplementary material for: Fly ash upcycling to functional silica nanomaterials: insights into synthetic strategies towards efficient adsorbents for water purification
Source: RSC Adv. 2026 Jan 2;16(1):362–9. doi: 10.1039/d5ra08626d (PMC12757929; doi:10.1039/d5ra08626d)
Supplement: RA-016-D5RA08626D-s001 [file RA-016-D5RA08626D-s001.pdf]

Supplementary Information (SI) for RSC Advances.
This journal is © The Royal Society of Chemistry 2025

Supplementary Information

Fly Ash Upcycling to Functional Silica Nanomaterials: Insights into Synthetic Strategies towards Efficient adsorbents for Water Purification

Miguel S. Ruiz[a], Cristian Tunsu[b], Fredric. G. Svensson[c], Ani Vardanyan[*a]

[a] Department of Molecular Sciences, Swedish University of Agricultural Sciences, P.O. Box 7015, 75007 Uppsala, Sweden

[b] EasyMining Services AB, Ultunaallén 2A, 756 51 Uppsala, Sweden

[c] Department of Materials Science and Engineering, The Ångström Laboratory, Uppsala University, Uppsala Box 35, 751 03, Sweden.

[*]Corresponding author-Dr. Ani Vardanyan, ani.vardanyan@slu.se, Department of Molecular Sciences, Swedish University of Agricultural Sciences, P.O. Box 7015, 75007 Uppsala, Sweden

Table S1. Elemental analysis of FA. The sample underwent microwave-assisted digestion in a mixture of nitric acid, hydrochloric acid, and hydrofluoric acid at elevated temperature. The total element content in the solution was then measured with Inductively Coupled Plasma Sector Field Mass Spectrometry (ICP-SFMS; Thermo Scientific Element XR)

**Element**	**Amount (mg/kg dry FA)**
**Al**	31900
**As**	16.6
**Ba**	984
**Be**	1.16
**Ca**	89700
**Cd**	2.55
**Co**	16.4
**Cr**	99.6
**Cu**	663
**Fe**	99600
**Hg**	0.0536
**K**	15200
**Mg**	21700
**Mn**	566
**Mo**	8.09
**Na**	7460
**Nb**	7.72
**Ni**	76.3
**P**	85000
**Pb**	142
**S**	3300
**Sb**	9.72
**Sc**	4.77
**Si**	165000
**Sn**	49.7
**Sr**	1510
**Ti**	4920
**V**	62.2
**W**	18.9
**Y**	15.3
**Zn**	2600
**Zr**	148
LOI 1000°C	1.52 %

Table S2. Elemental analysis of FA residue after acid treatment. The sample underwent microwave-assisted digestion in a mixture of nitric acid, hydrochloric acid, and hydrofluoric acid at elevated temperature. The total element content in the solution was then measured with Inductively Coupled Plasma Sector Field Mass Spectrometry (ICP-SFMS; Thermo Scientific Element XR)

**Element**	**Amount (mg/kg dry FA)**
**Al**	38400
**As**	3.16
**Ba**	2350
**Be**	1.08
**Ca**	33400
**Cd**	0.145
**Co**	4.1
**Cr**	123
**Cu**	132
**Fe**	15800
**Hg**	<0.05
**K**	21900
**Mg**	3550
**Mn**	119
**Mo**	1.66
**Na**	8850
**Nb**	14.4
**Ni**	20.2
**P**	7810
**Pb**	47.2
**S**	1620
**Sb**	9.15
**Sc**	2.79
**Si**	343000
**Sn**	19.4
**Sr**	550
**Ti**	9350
**V**	37
**W**	19
**Y**	9.67
**Zn**	316
**Zr**	238
LOI 1000°C	2.84 %

Table S3. FA composition before and after Si extraction measured by EDS analysis

	FA	Si extracted FA
**Element**	**Atomic %**	**Atomic %**
O	58.88	57.12
Si	17.96	7.63
K	0.99	0.69
Ti	0.74	1.22
Fe	0.68	1.12
P	0.55	0.02
Na	0.48	13.47
Mg	0.30	0.19
Al	2.41	1.67
Ca	0.14	0.14
C	15.03	15.86
N	1.71	0.85
S	0.11	0.00
Total	100.00	100.00

Table S4. Elemental composition of SSS measured by EDS analysis. The SSS solution was dried on the carbon tape prior the measurement

	SSS solution
**Element**	**Atomic %**
C	23.87
O	53.80
Na	20.54
Si	1.29
P	0.15
N	0.34
Total	100.00

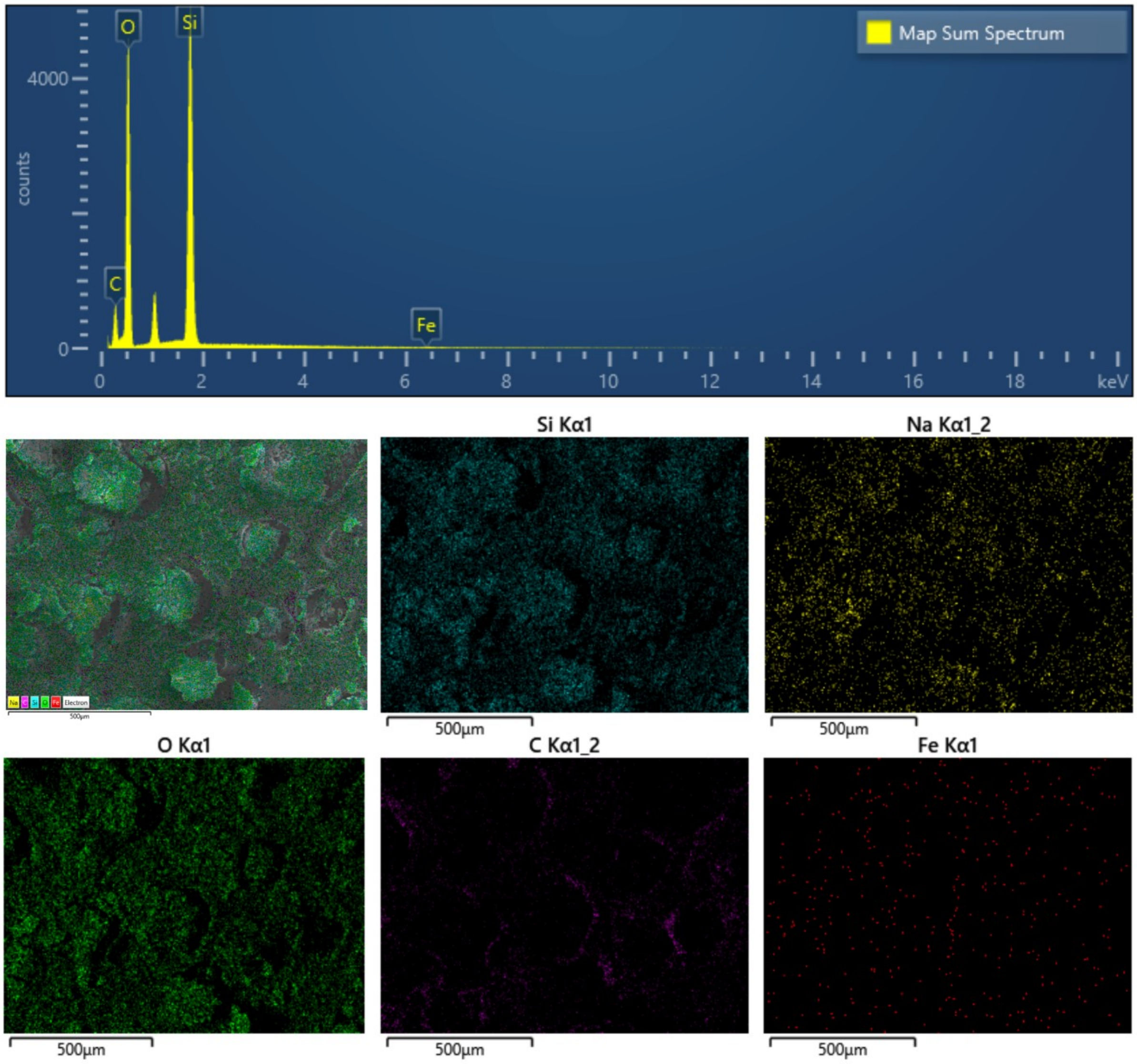

Fig. S1 EDS analysis of dense SiO_2 NPs derived from FA

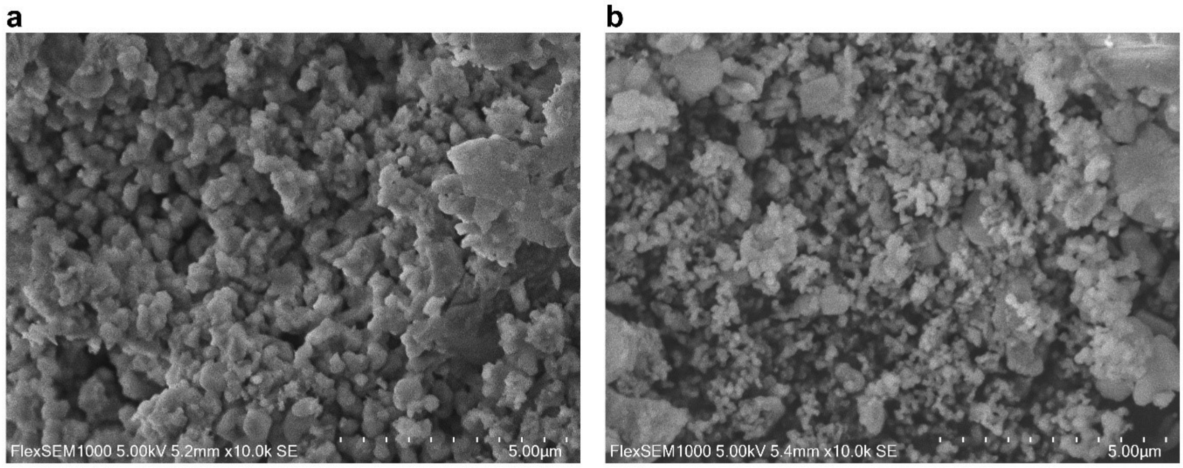

Fig. S2 SEM images of synthesized M_SiO_2 NPs (a) and 1 pot_M_SiO_2 (b)

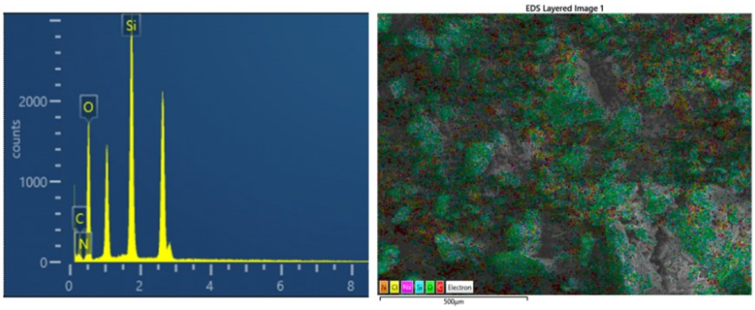

Map Sum Spectrum Element	Atomic %
C	31.99
O	41.81
Na	7.79
Si	10.23
Cl	8.18
N	0.00
Total	100.00

Fig. S3 EDS analysis of M_SiO2 NPs derived from FA

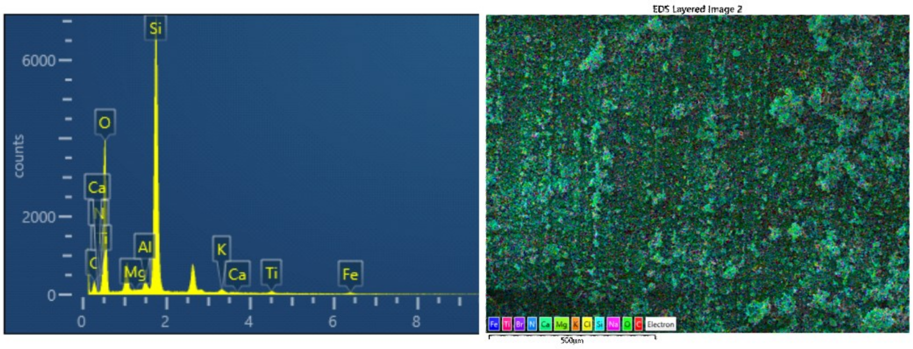

Map Sum Spectrum Element	Atomic %
C	23.24
O	52.37
Si	15.06
Cl	1.87
Na	2.78
K	0.26
Al	0.48
Ti	0.22
Fe	0.24
Mg	0.00
Ca	0.01
N	3.47
Total	100.00

Fig. S4 EDS analysis of 1 pot_M_SiO2 NPs derived from FA

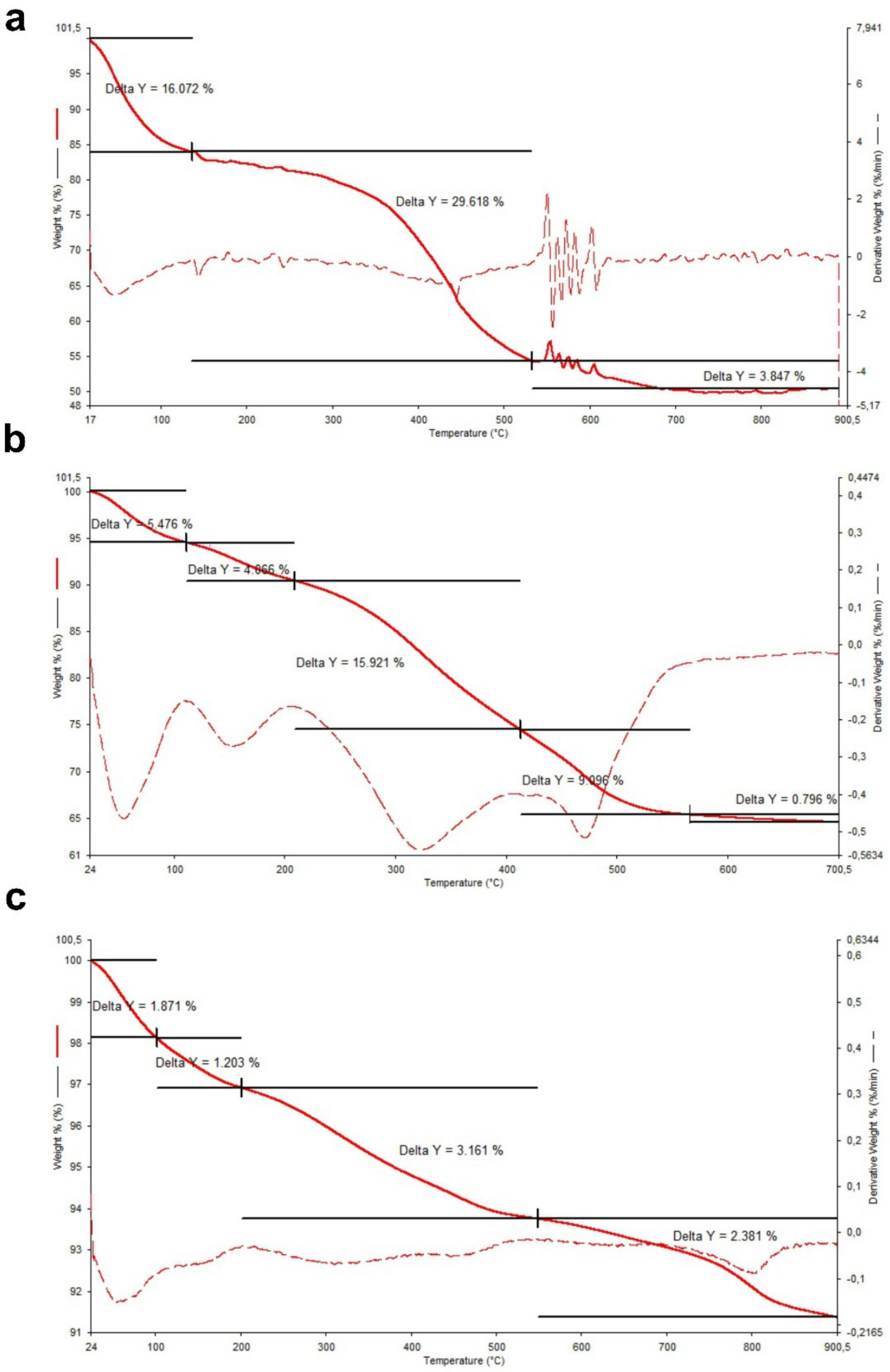

Fig. S5 TGA curves of synthesized SiO_2 nanomaterials grafted with TMSPDETA ligand: (a) M_SiO_2, (b) D_SiO_2 and (c) 1 pot_M_SiO_2 NPs

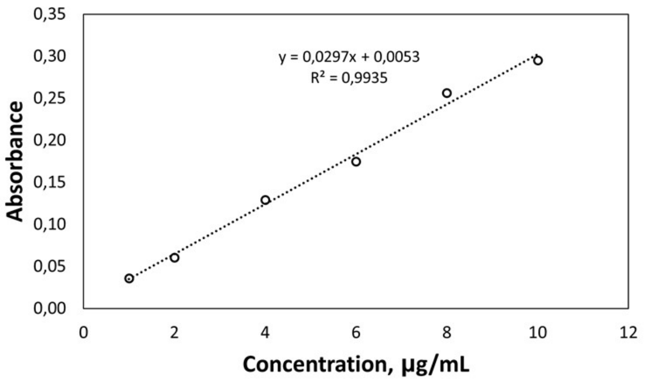

Fig. S6 Calibration curve DFC measured by UV-Vis with DFC standard concentrations varying between 1-10 μg/mL

Table S5. Amount of grafted TMSPDETA ligand on synthesized SiO_2 NPs

Sample	Grafted TMSPDETA (mmol/g)
D_SiO_2	0.94
M_SiO_2	1.12
1 pot_M_SiO_2	0.2

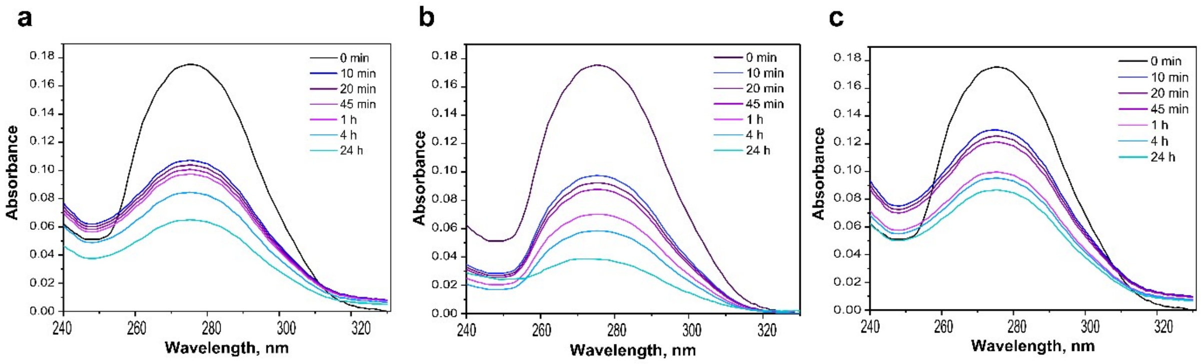

Fig. S7 Adsorption kinetics of DFC on SiO_2 nanomaterials: (A) D_SiO_2, (B) M_SiO_2 and (C) 1 pot_M_SiO_2 NPs
